# 

**Published:** 1897-06

**Authors:** 


### Communicated.

### THE NATIONAL ASSOCIATION OF DENTAL EXAMINERS.

The fourteenth annual session will be held in the parlors of the "Hotel Chamberlin," Old Point Comfort, Va., Friday morning, July 30th, 1897, ten o'clock, and continue in session (with the exception of Sunday, August 1st) until adjournment. Matters of the greatest importance to the Association will come before this meeting, and it is hoped every member of all the various State Boards will be present.

"Hotel Chamberlain" and "Hygeia Hotel" rates will be $2.50 per day, two in a room; $3.50 per day, one in a room.

Fare from all points on all the railroads will be one and a third; take your receipt from ticket agent when purchasing ticket. The Old Dominion Steamship Co., Pier 26, North River, New York, will sell excursion tickets, including meals and berth, for $11.20, sailing Thursday July 29th, 3 p. m.

CHARLES A. MEEKER, D. D. S., Sec'y,
29 Fulton street, Newark, N. J.

---

### AMERICAN DENTAL ASSOCIATION.

The thirty seventh annual session of the American Dental Association will be held at Old Point Comfort, Va,, commencing at 10 A. M., on Tuesday, August 3rd, 1897.

GEO. H. CUSHING,
Recording Secretary.

---

### Editorial, Etc.

### To Readers and Correspondents.

Communications intended for publication, and exchange journals and papers, should be addressed to the Editor of The American Journal of Dental Science, No. 845 N. Eutaw St. Baltimore, Md. All letters relating to the business of the Journal, or containing cash remittances, to be directed to Snowden & Cowman Mfg. Co., 9 W. Fayette Street, Baltimore, Md. Articles for publication should be received by the Editor at least two weeks previous to the first of the month on which the number in which it is designed for them to appear is issued.

☞ The American Journal of Dental Science will contain fifty pages of reading matter in each number, making a volume of about Six Hundred pages,

EXCLUSIVE OF ADVERTISEMENTS.

